# First trimester medication use in pregnancy in Cameroon: a multi-hospital survey

**DOI:** 10.1186/s12884-018-2081-x

**Published:** 2018-11-20

**Authors:** Aminkeng Zawuo Leke, Helen Dolk, Maria Loane, Karen Casson, Nkwati Michel Maboh, Susan Etta Maeya, Lerry Dibo Ndumbe, Pauline Bessem Nyenti, Obale Armstrong, Derick Etiendem

**Affiliations:** 1 Department of Nursing, School of Health Sciences, Biaka University Institute of Buea-Cameroon, PO BOX 77, Buea, Cameroon; 2 Centre for Maternal, Fetal and Infant Research, Institute for Nursing and Health Research, Ulster University, Shore Rd Newtownabbey, BT370QB Ulster, Ireland; 3 Office of the Deputy Vice Chancellor i/c Research/Cooperation/Quality, Biaka Universit Institute of Buea, PO Box 77-SWR, Buea, Cameroon

**Keywords:** Medication, pregnancy, drug safety, drug use, pharmacoepidemiology, determinants, pharmacovigilance, Cameroon

## Abstract

**Background:**

There is a paucity of epidemiological data on medication use in pregnancy in Cameroon.

**Methods:**

Between March and August 2015, 795 pregnant women attending 8 urban and 12 rural hospitals in Cameroon for antenatal (ANC) or other care were interviewed on first trimester medication use using structured questionnaires. Multivariate logistic regression was used to analyse the association of 18 sociodemographic factors with medication use.

**Results:**

A total of 582 (73.2%) women took at least one orthodox (Western) medication during the first trimester, 543 (68.3%) women a non-pregnancy related orthodox medication, and 336 (42.3%)women a pregnancy related orthodox medication. 44% of the women took anti-infectives including antimalarials (33.6%) and antibiotics (20.8%).The other most common medications were analgesics (48.8%) and antianaemias (38.6%). Sulfadoxine/pyrimethamine, contraindicated in the first trimester of pregnancy, was the most commonly used antimalarial(13% of women).0.2% of women reported antiretroviral use. Almost 80% of all orthodox medications consumed by women were purchased from the hospital. 12.8% of the women self-prescribed. Health unit and early gestational age at ANC booking were consistent determinants of prescribing of non-pregnancy related, pregnancy related and anti-infective medications. Illness and opinion on the safety of orthodox medications were determinants of the use of non-pregnancy related medications and anti-infectives. Age and parity were associated only with non-pregnancy related medications.

**Conclusion:**

This study has confirmed the observations of studies across Africa indicating the increasing use of medications during pregnancy. This is an indication that access to medicine is improving and more emphasis now must be placed on medication safety systems targeting pregnant women, especially during the first trimester when the risk of teratogenicity is highest.

**Electronic supplementary material:**

The online version of this article (10.1186/s12884-018-2081-x) contains supplementary material, which is available to authorized users.

## Background

Pregnant women are excluded from clinical trials for medication efficacy, since the fetus is particularly vulnerable to adverse effects. This means that at the time of marketing, there is no information on human safety in pregnancy. Since premarketing teratogenicity testing in animals is not enough to predict risks in humans, for the majority of medications the safety of their use during pregnancy is still not clearly defined [1]. Safety information needed by women and clinicians in order to be able to weigh the risks and benefits of different treatment options must therefore come from post-marketing pharmacoepidemiological studies. Within this context, epidemiological studies investigating medication use and safety in pregnancy are of paramount importance.

Minimal attention has been given to the subject of medication use and safety during pregnancy in developing countries which, ironically, have higher rates of adverse maternal/fetal outcomes, greater drug quality control problems compared to developed countries, and less resources to care for the babies born with problems relating to their exposure in utero. In Cameroon for example, WHO (2017) [2] statistics showed a maternal mortality rate of 596/100.000 (ranking 14^th^ in the world) and a neonatal mortality rate of 87.9/1000 (21 times higher than that in the UK). Drug safety constraints in Sub-Saharan Africa countries like Cameroon include: lack of strict control mechanisms as to which drugs are accepted or not accepted into the country, hence circulation of substandard, counterfeit, and contaminated drugs [3]; lack of stringent prescription regulations, e.g in Cameroon nurses are allowed to prescribe highly specialized medications such as psychotropics [4]; high rate of self-medication; availability of most medications over-the-counter; and common use of traditional herbs [5] which are believed by many users to be more potent than orthodox medicine and to have no adverse effects [6]. In such a system, there are high chances of pregnant women consuming potentially teratogenic medications, including those categorized as contraindicated in pregnancy.

While data on medication use are critical in understanding the depth of medication safety concerns, understanding the determinants of medication use can provide valuable insights as to which subgroup of women could be at higher risk of consuming potentially teratogenic medications [7]. Such data could be valuable to public health authorities in designing targeted interventions. The few studies conducted in Africa have found setting type (urban/rural), history of negative pregnancy outcome, illness during pregnancy, gravidity, pregnancy planning and level of education of healthcare provider to be significant predictors of medication use [8, 9]. As determinants of medication use could vary from context to context [10], it is difficult to extrapolate data across different contexts.

This medication use survey provides data on the prevalence, types and determinants of orthodox medication use by pregnant women in Cameroon, the first survey of this population. Results regarding herbal or traditional medication use will be reported separately.

## Methods

This cross sectional hospital based medication use survey was conducted during a six month period (March to August 2015) in twenty hospitals across rural and urban settings of the southwest region of Cameroon. Taking into consideration the total number of live births in South West Cameroon for 2013 (12,861 births of approximately 6,687 urban and 6,174 rural), a 6 months data collection period, and a 50% response distribution (worst case scenario), maximum sample size needed was estimated at approximately 374 for both urban and rural strata.

We used a two stage cluster sampling technique: in the first step, eligible hospitals were randomly selected and in the second step, all eligible women within the selected hospitals attending during the study period were invited to participate.

Both private and government hospitals were eligible to participate if they had an annual delivery rate of over one hundred and two hundred for rural and urban settings respectively. Out of forty-one eligible hospitals (twenty-two urban and nineteen rural), 20 were randomly selected.

All pregnant women attending the selected hospitals on the days the researchers were in attendance (registered for antenatal clinics or not) were eligible to participate. To limit recall bias and to target the period within first trimester, only women with a gestation of three to seven months were eligible to participate in the survey. The pregnant women were recruited as they came for antenatal visits or in a small number of cases, for hospital consultation. Out of eight hundred and seventeen eligible women approached, seven hundred and ninety-five agreed to participate in the study (97.3% response rate).The observed distribution of the women into urban (55.2%) and rural (44.8%) settings of residence matched the expected distribution in the general population. Similarly, the proportion of women sampled in each health district within the data collection period was comparable to that of 2013 delivery data.

Existing literature on medication use and safety were reviewed to facilitate the design of a questionnaire to be used by interviewers (Additional file 1). The questionnaire was designed to facilitate recall (e.g. the woman had to define the three months of her first trimester (exposure period of interest) prior to completing the section on medication exposure; the section on medication exposure was followed by a section on first trimester illnesses so as to validate data on exposure given (for example, if a woman reported having malaria during the first trimester, one would verify whether she reported taking an antimalarial in the previous section on medication exposure).

A picture guide of orthodox and traditional medications was developed to facilitate recall. In Cameroon, patients have individual hospital books which they bring along during hospital visits. Antenatal care (ANC) files kept in the hospitals contain data only on medications prescribed during routine ANC visits. Hence medication data for other hospital visits could only be obtained from hospital books. When available, the data collectors used antenatal files and the hospital books to complement and validate data obtained from interviews. In a sub-study involving 84 participants to evaluate the relevance of using the hospital books (Table 1), we observed that 10.3% of the exposures would have been missed without the use of hospital books.

**Table 1:** Comparison of source of medication exposure (N=84 Women)^a^

Medication mentioned in interview but not found in hospital book n (%)	Medications found in hospital book but not mentioned during interview n (%)	Medications mentioned during interviews and also found in hospital book n (%)	Total n (%)
51 (16.0)	33 (10.3)	236 (73.8)	320 (100)

^a^ Excluding those without a hospital book and those that did not take medications according to the hospital book

In order to ensure standardized collection of data, eight nurses working in the areas of research and education were trained as data collectors and provided with a guidance note to assist them during data collection. A pilot study enabled the data collectors to feed back to adjust the questionnaire and various aspects of the data collection process.

Actual data collection took place from the months of March to August 2015. Using the predesigned questionnaire, the data collectors conducted one-on-one interviews for consented women in private rooms of the hospital to obtain data on first trimester medication exposure.

Following the approach of Baraka et al (2014) [10], orthodox medications were grouped as pregnancy related medications and non-pregnancy related medications. Pregnancy related medications were defined as routine medications taken not for ill-health, but to support the health of the mother and the developing fetus. These included anti-anemias, mineral supplements and vitamins.

The Anatomical Therapeutic Chemical Classification System of the WHO was used to classify drugs into therapeutic classes. Drugs were also classified according to the old version of United States Food and Drug Administration (FDA) pregnancy risk classification (A, B, C, D or X; see foot note in Fig 6). The FDA classification of each drug was verified from various sources including normal Google search, the Internet Drug Index (RxList) and Drugs .com. Drugs for which no FDA class could be obtained were classified as category “U”.

Epi-info 3.1 was used for data entry and cleaning while all data analyses were conducted using SPSS version 22. Prevalence of medication use was determined by dividing the number of women who took at least one medication by the total number of participating women. Differences in the prevalence of medication use within categorical variables were tested using the Pearson Chi-squared test of independence with significance level set at 0.05. Multivariatelogistic regression was used to identify the determinants of medication use. Using backward conditional logistic regression, all the variables were initially included in the model. Then, variables were removed from the model based on significance level set at 0.10, if their removal did not significantly worsen the overall prediction of the model [11]. Variables (all categorical) entered into the model were: health unit (individual primary or secondary/tertiary healthcare facility), setting of hospital (urban/rural), maternal age (13-17 years, 18-25 years, 26-35 years and 36-45 years), marital status, highest level of education attained, living conditions, level of alcohol consumption, gravidity, parity, previous pregnancy termination, gestational age at interview, gestational age at first booking, pregnancy planning, gestational age at pregnancy awareness, opinion on the safety of orthodox medication, safety advice and illness during first trimester. We investigated determinants for general orthodox medication use, pregnancy related medication use, and anti-infectives use. Results were reported as adjusted odds ratios and 95%CI. Determinants of medication use were defined as those variables retained in the final logistic regression model.

## Results

A total of twenty hospitals (eight urban and twelve rural) were involved in this study with a sample size of seven hundred and ninety-five participants (four hundred and thirty-nine urban and three hundred and fifty-six rural, Table 2). Table 2 presents data on the general characteristics of the women and their association with medication intake.

**Table 2:** Factors associated with maternal use of orthodox medication (N=795)

Factor	Total	At least one orthodox medication	Chi Sq.	df	P-value
	n (%)	n (%)			
	795(100)	582(73.2)			
Health unit			135.0	19	0.000
A^r,p^	43(5.4)	21 (48.8)			
B^r,g^	37(4.7)	30 (81.1)			
C^r,g^	29(3.6)	23 (79.3)			
D^r,g^	22(2.8)	20 (90.9)			
E^u,p^	29(3.6)	27 (93.1)			
F^u,g^	39(4.9)	23 (59.0)			
G^r,g^	36(4.5)	19 (52.8)			
H^r,g^	35(4.4)	28 (80.0)			
I^r,g^	26(3.3)	22 (84.6)			
J^u,g^	44(5.5)	32 (72.7)			
K^u,p^	50(6.3)	49 (98.0)			
L^u,g^	15(1.9)	14 (93.3)			
M^u,g^	67(8.4)	52 (77.6)			
N^u,p^	18(2.3)	17 (94.4)			
O^r,g^	108(13.6)	45 (41.7)			
P^r,g^	21(2.6)	18 (85.7)			
Q^r,g^	45(5.7)	42 (93.3)			
R^r,g^	56(7.0)	39 (69.6)			
S^r,g^	49(6.2)	36 (73.5)			
T^u,g^	26(3.3)	25 (96.2)			
Health Districts			43.9	6	0.000
Fontem^a^	43(5.4)	21 (48.8)			
Mbonge	66(8.3)	53 (80.3)			
Kumba	90(11.3)	70 (77.8)			
Muyuka	97(12.2)	69 (71.1)			
Buea^a^	194(24.4)	164 (84.5)			
Tiko^a^	174(21.9)	105 (60.3)			
Limbe	131(16.5)	100 (76.3)			
Setting type			1.4	1	0.235
Urban	439(55.2)	314 (71.5)			
Rural	356(44.8)	268 (75.3)			
Age (years)			1.9	3	0.594
13-17	41(5.2)	24 (58.5)			
18-25	380(47.8)	285 (75.0)			
26-35	335(42.1)	243 (72.5)			
36-45	39(4.9)	30 (76.9)			
Marital status		()	2.5	4	0.645
Married	486(61.1)	352 (72.4)			
Divorced	6(0.8)	4 (66.7)			
Engaged	64(8.1)	44 (68.8)			
Cohabitating (No formal engagement)	68(8.6)	54 (79.4)			
Single	171(21.5)	128 (74.9)			
Level of Education			33.9	4	0.000
Never went to school	19(2.4)	13 (68.4)			
Primary^a^	205(25.8)	123 (60.0)			
Secondary	329(41.4)	245 (74.5)			
High School	129(16.2)	101 (78.3)			
University/Professional^a^	113(14.2)	100 (88.5)			
Living condition			13.8	2	0.001
House with Pit /external toilet^a^	597(75.1)	417 (69.8)			
Renting self-contained studio	104(13.1)	86 (82.7)			
Renting or Own a self-contained House	94(11.8)	79 (84.0)			
Level of Alcohol consumption in pregnancy			0.4	3	0.937
Do not drink Alcohol	362(45.5)	264 (72.9)			
Drink Occasionally	324(40.8)	237 (73.1)			
1-2 bottles of beer/glass of wine a week	86(10.8)	65 (75.6)			
Greater than 1 bottles of beer/glass of wine a day	23(2.9)	16 (69.6)			
Gravidity			4.6	4	0.332
1	293(36.9)	221 (75.4)			
2	197(24.8)	145 (73.6)			
3	152(19.1)	114 (75.0)			
4	94(11.8)	64 (68.1)			
≥5	59(7.4)	38 (64.4)			
Parity			7.5	3	0.057
0	323(40.6)	236 (73.1)			
1	202(25.4)	155 (76.7)			
2	145(18.2)	111 (76.6)			
≥3	125(15.7)	80 (64.0)			
Terminations/miscarriages			1.9	2	0.385
0	424(53.3)	308 (72.6)			
1	56(7.0)	40 (71.4)			
2	22(2.8)	13 (59.1)			
GA at interview			7.6	3	0.056
4th month	212(26.7)	168 (79.2)			
5th month	230(28.9)	171 (74.3)			
6th month	294(37.0)	203 (69.0)			
7th month	59(7.4)	40 (67.8)			
GA at ANC booking			72.8	3	0.000
< 13 weeks^a^	199(25.0)	184 (92.5)			
13 - 24 weeks^a^	529(66.5)	369 (69.8)			
25 - 28 weeks	23(2.9)	12 (52.2)			
Not registered for ANC^a^	44(5.5)	17 (38.6)			
Pregnancy planning		()	2.0	2	0.372
Yes	474(59.6)	353 (74.5)			
No	300(37.7)	216 (72.0)			
Unknown	21(2.6)	13 (61.9)			
GA of pregnancy awareness			1.1	3	0.749
1st month	476(59.9)	346 (72.7)			
2nd month	131(16.5)	97 (74.0)			
≥3th month	32(4.0)	26 (81.3)			
Unknown	156(19.6)	113 (72.4)			
Number of diseases/ailments			162.6	6	0.000
0^a^	139(17.5)	52 (37.4)			
1^a^	152(19.1)	90 (59.2)			
2	142(17.9)	115 (81.0)			
3^a^	116(14.6)	104 (89.7)			
4^a^	108(13.6)	100 (92.6)			
5^a^	67(8.4)	61 (91.0)			
≥6	71(8.9)	60 (84.5)			
Number (types) of acute conditions			31.8	6	0.000
0	146(18.4)	56 (38.4)			
1^a^	154(19.4)	50 (32.5)			
2	145(18.2)	71 (49.0)			
3^a^	129(16.2)	74 (57.4)			
4^a^	106(13.3)	62 (58.5)			
5	61(7.7)	23 (37.7)			
≥6	54(6.8)	20 (37.0)			
Number (types) of chronic conditions			7.7	2	0.023
0	696(87.5)	300 (43.1)			
1^a^	89(11.2)	52 (58.4)			
≥2	10(1.3)	4 (40.0)			
Season			0.0	1	0.933
Dry	738 (92.8)	540 (73.2)			
Rainy	57(7.2)	42 (73.7)			
Opinion on safety of Orthodox medicationduring pregnancy			1.3	3	0.026
Yes, it is always safe	551(69.3)	395 (71.7)			
Yes, safe but depends^a^	157(19.7)	129 (82.2)			
No, never safe	21(2.6)	15 (71.4)			
I don't know	66(8.3)	43 (65.2)			
Participant received medication safety advice during current pregnancy			18.4	2	0.000
Yes^a^	468(58.9)	369 (78.8)			
No^a^	287(36.1)	187 (65.2)			
Can't remember	40(5.0)	26 (65.0)			

^a^Statistical significance as calculated from the adjusted standardised residuals

*r* Rural hospital, *u* Urban hospital, *p* Private hospital, *g* Government hopsital

*Chi Sq* Chi-squared value based on test of independence, *df* degrees of freedom

P = 0.05 indicates a statistically significant association based on the Chi-squared test of independence

The age of the women ranged from thirteen to forty-five years, with the highest academic level for most being primary (25.8%) or secondary (41.4%) (Table 2).

Three quarters (75.1%) of the women lived in houses with pit/external toilets. About a third (36.9%) of the women were in their first pregnancy. Only 25% of the women registered for ANC within the first trimester. Some women (5.5%) although they had visited the hospital had not registered for ANC.

Based on the definition of pregnancy planning as used in this study, 37.7% of the women did not engage in sexual intercourse with the intention of getting pregnant. However, the majority (59.9%) of the women said they were aware of their pregnancy within the first month of gestation.

More than half (54.5%) reported they consume alcohol in pregnancy.

### First trimester orthodox medication use

**Fig. 1:**
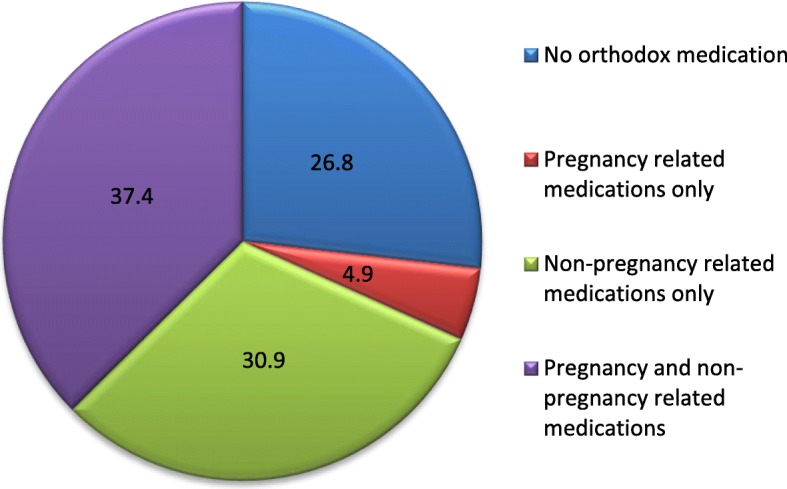
Distribution of pregnancy and non - pregnancy related orthodox medications (N=795)

Almost three quarters (73.2%, n=582) women took at least one orthodox medicationduring the first trimester of pregnancy (Table 2). Out of the five hundred and eighty-two women who took an orthodox medication,30.9% took a non-pregnancy related orthodox medication only and 4.9% took a pregnancy related orthodox medication only (Fig. 1), most women taking both categories.

**Fig. 2:**
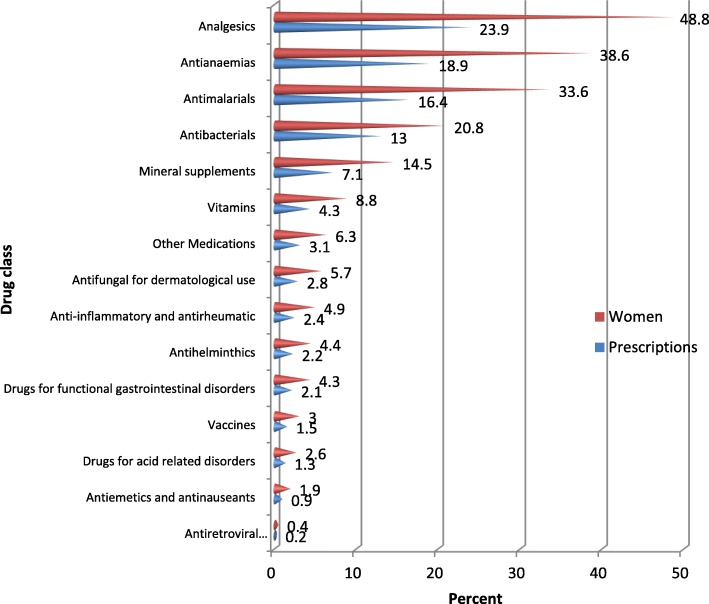
Distribution of reported orthodox medications taken by class (N=795 women)

Fifteen different classes of medications were reported by the women (Fig. 2). Analgesics, antianaemias, antimalarials, antibacterials and mineral supplements were the top five classes of medications consumed. Ferrous sulphate (Fefol) was the most commonly used iron and folic acid prophylactic supplement containing 65mg of elemental iron and 0.4mg of folic acid.

**Fig. 3:**
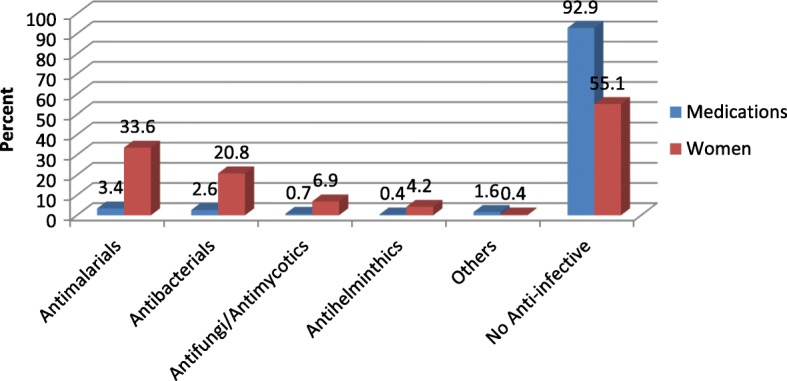
Frequency of consumption of class of anti-infectives (N=795 women)

**Fig. 4:**
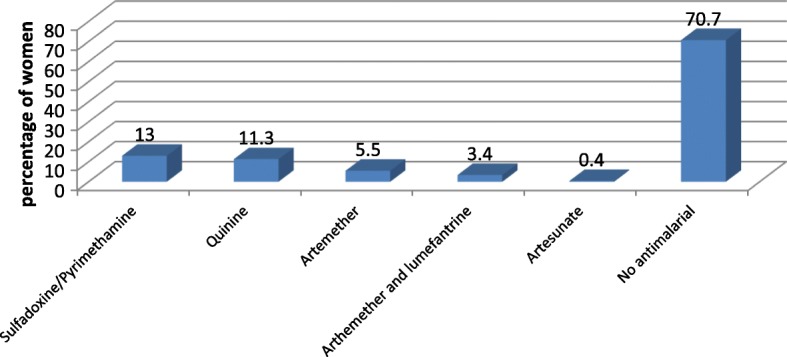
Exposure of women to antimalarials (N=795). NB 30% of women who took a non sulfadoxine/pyrimethamineantimalaria did not report any malaria illness

**Fig. 5:**
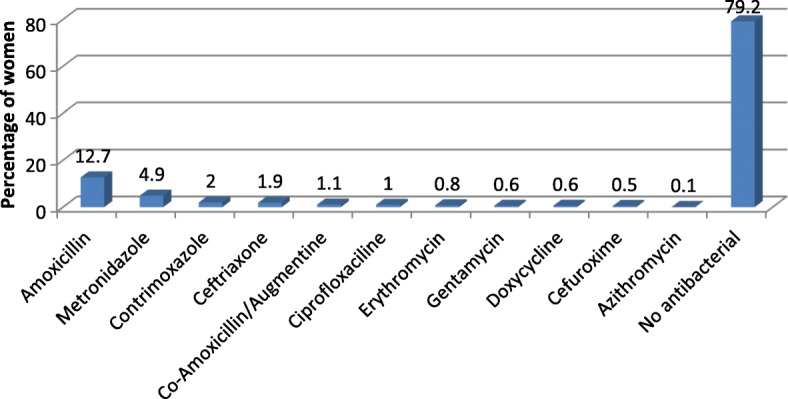
Exposure of women to antibacterials

With the high burden of infectious diseases in Cameroon, we paid particular attention to the anti-infectives data. 44.9% of women took one or more anti-infectives. Antimalarials (33.6%) followed by antibacterials (20.8%) were the most commonly consumed categories of anti-infectives (Fig. 3). Sulfadoxine/pyrimethamine and Quinine; and Amoxicillin and Metronidazole were the most commonly consumed antimalarials and antibacterials respectively (Figs. 4 and 5).

### FDA pregnancy risk classification of drugs

**Fig. 6:**
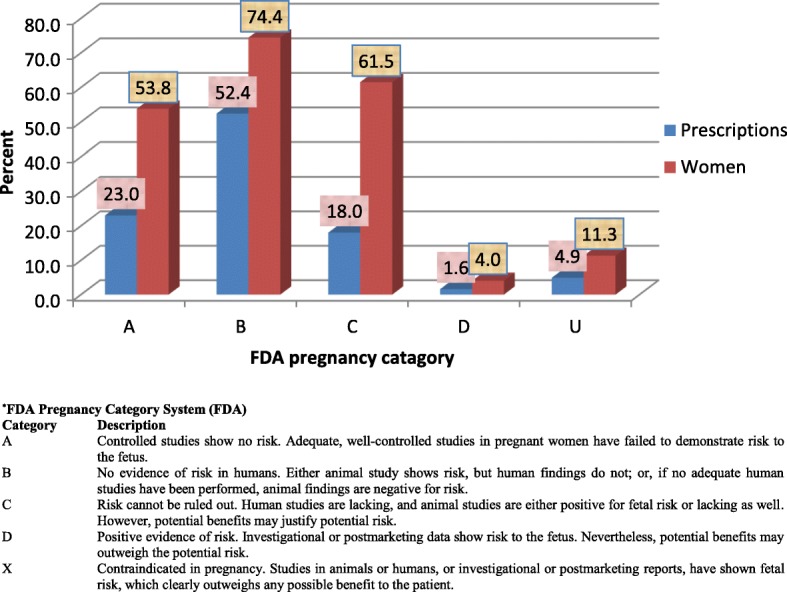
Othodox medication by FDA pregnancy categories (N=582) (based only on women who took orthodox medication, FDA category U=Undefined)

Most of the women took a category B (74.4%) or C (61.5%) medication (Fig. 6). A potentially teratogenic medication was defined as those belonging to FDA categories C, D or X. There was no intake of a category X medication. However, out of the five hundred and eighty-two women who took orthodox medications, a large proportion (65.5%) took a category C and/or D drug. Projected to the entire sample of 795, this corresponded to 48% of women. Drugs belonging to category D included: phenobarbital (one exposure), ibuprofen (nineteen exposures), diclofenac (eleven exposures), fluconazole (five exposures), and magnesium (three exposures). Antimalarials constituted the bulk of this proportion as they are mainly classified as FDA category C. When antimalarials were excluded, the overall consumption of potentially teratogenic medications reduced to 17% of the total study sample.

### Sources and prescribers of orthodox medications

Almost 80% of all orthodox medications consumed by women were purchased from the hospital. Other sources were regular market (10.1%), commercial pharmacy (6.1%), medicine store (5.1%) and unknown (0.9%).

The majority (82%) of women took medications based on prescription from their healthcare provider in the hospital, but 12.8% of the women self-prescribed at least one medication, and 3.7% took at least one medication recommended by a friend or relative.

### Determinants of orthodox medication use

Determinants of use were determined for three groups of orthodox medications: non-pregnancy related (Table 3), pregnancy related (Table 4) and anti-infectives (Table 5). Health unit and gestational age at ANC booking were constant detrminants across the three categories. Women who booked for ANC early (within first trimester) were more likely to take any of the three groups of orthodox medications ((AOR: 2.6; CI=1.3-5.2); (AOR: 1.4; CI=0.9-2.4); (AOR: 2.7; CI=1.6-4.5); for non-pregnancy related medications, pregnancy related medications and anti-infectives respectively). The variation in medication use by Health Unit was wide (Table 2) and persisted after adjusting for other factors (Tables 3, 4, 5, see Additional file 2 for complete table showing all health units). Parity and age were associated with non-pregnancy related orthodox medications (Table 3). Younger women (<17years) were less likely (AOR: 0.1; CI=0.0-0.8), while multiparous women were more likely (e.g. those with a parity of 2 (AOR: 4.0; CI=1.3-12.7)) to take a non-pregnancy related orthodox medication. High level of education, receipt of safety advice, and rural setting type were associated with more use of pregnancy related medication (Table 4). Illness and opinion on the safety of orthodox medications were associated with use of seen as predictors for non-pregnancy related medications and anti-infectives, but not pregnancy related medications.

**Table 3:** Logistic regression model for predicting consumption of non-pregnancy related orthodox medication (N=795)

Variables	Crude model					Adjusted model			
	Exposed (%)	OR	95% C.I		P-value	OR	95% C.I		P-value
			Lower	Upper			Lower	Upper	
Health unit(20)^b^					0.000^a^				0.000^a^
Maternal age (years)					0.095^a^				0.049^a^
18-25	71.3	1	-	-	-	1	-	-	ref
13-17	53.7	0.5	0.2	0.9		0.1	0.0	0.8	
26-35	66.3	0.8	0.6	1.1		0.6	0.4	1.1	
36-45	71.8	1.0	0.5	2.1		1.3	0.5	3.8	
Parity					0.107^a^				0.006^a^
0	68.7	1	-	-	-	1	-	-	ref
1	70.8	1.1	0.8	1.6		2.9	1.0	8.6	
2	71.7	1.2	0.8	1.8		4.0	1.3	12.7	
≥3	59.2	0.7	0.4	1.0		1.5	0.5	4.9	
Gestational age at ANC booking					0.000^a^				0.001^a^
13 - 24 weeks	65.8	1	-	-	-	1	-	-	ref
< 13 weeks	83.9	2.7	1.8	4.1		2.6	1.3	5.2	
25 - 28 weeks	52.2	0.6	0.2	1.3		1.6	0.4	6.4	
Not registered for ANC	36.4	0.3	0.2	0.6		0.3	0.1	0.8	
Illness during first trimester					0.000^a^				0.000^a^
No	32.8	1	-	-	-	1	-	-	ref
Yes	79.4	7.9	5.5	11.3		11.1	6.0	20.4	
Opinion on safety of orthodox medication					0.180^a^				0.078^a^
Yes, it is always safe	67.2	1	-	-	-	1	-	-	ref
Yes, safe but depends	75.2	1.5	1.0	2.2		0.7	0.4	1.3	
No, never safe	66.7	1.0	0.4	2.5		0.1	0.0	0.8	
I don't know	62.1	0.8	0.5	1.4		0.5	0.2	1.3	

^a^ Overall statistical significance of variable within model

^b^ See Additional file 2 for complete table showing statistics for all health units

*OR* Odds ratio, *CI* Confidence interval, *r* Rural hospital, *u* Urban hospital, *p* Private hospital, *g* Government hopsital

**Table 4:** Logistic regression model for predicting consumption of pregnancy related orthodox medication (N=795)

Variables	Crude model					Adjusted model			
	Exposed (%)	OR	95% C.I		P-value	OR	95% C.I		P-value
			Lower	Upper			Lower	Upper	
Health unit(20)^b^					0.000^a^				0.000^a^
Highest level of Education					0.000^a^				0.072^a^
Secondary	37.4	1	-	-	-	1	-	-	ref
Never went to school	38.0	1.0	0.7	1.5		0.9	0.5	1.4	
Primary	26.3	0.6	0.2	1.7		0.2	0.0	0.6	
High School	45.0	1.4	0.9	2.1		1.0	0.5	1.7	
University/Professional	63.7	2.9	1.9	4.6		1.4	0.7	3.0	
Gestational age at ANC booking					-				0.004^a^
13 - 24 weeks	39.5	1	-	-	0.000^a^	1	-	-	ref
< 13 weeks	59.8	2.3	1.6	3.2		1.4	0.9	2.4	
25 - 28 weeks	8.7	0.1	0.0	0.6		0.4	0.1	1.9	
Not registered for ANC	13.6	0.2	0.1	0.6		0.2	0.1	0.6	
Participant received safety advice					0.001^a^				0.057^a^
Yes	47.6	1	-	-	-	-	-	-	ref
No	34.5	0.6	0.4	0.8		0.6	0.4	0.9	
Can't remember	35.0	0.6	0.3	1.2		0.7	0.2	2.1	
Setting type					0.609^a^				0.042^a^
Urban	41.5	1	-	-	-	1	-	-	ref
Rural	43,3	1.1	0.8	1.4		3.3	1.0	10.2	

^a^Overall statistical significance of variable within model

^b^ See Additional file 2 for complete table showing statistics for all health units

*OR* Odds ratio, *CI* Confidence interval, *r* Rural hospital, *u* Urban hospital, *p* Private hospital, *g* Government hopsital

**Table 5:** Logistic regression model for predicting consumption of anti-infectives (N=795)

	Crude model					Adjusted model			
Variables	Exposed (%)	OR	95% C.I		P-value	OR	95% C.I		P-value
			Lower	Upper			Lower	Upper	
Health unit(20)^b^					0.000^a^				0.001^a^
Gestational age at ANC booking					0.00^a^				0.000^a^
13 - 24 weeks	42.2	1	-	-	-	1	-	-	ref
< 13 weeks	62.8	2.3	1.7			2.7	1.6	4.5	
25 - 28 weeks	17.4	0.3	0.1			0.6	0.1	2.4	
Not registered for ANC	11.4	0.2	0.1			0.3	0.1	0.9	
Illness during first trimester									
No	20.6	-	-	-	-	-	-	-	ref
Yes	52.5	4.2	2.9	6.3	0.000^a^	3.7	2.1	6.4	0.000^a^
Opinion on safety of orthodox medication					0.098^a^				0.056^a^
Yes, it is always safe	46.6	1	-	-	-	1	-	-	ref
Yes, safe but depends	45.9	1.0	0.7	1.4		0.6	0.4	1.1	
No, never safe	33.3	0.6	0.2	1.4		0.2	0.0	1.2	
I don't know	31.8	0.5	0.3	0.9		0.4	0.2	1.0	

^a^Overall statistical significance of variable within model

^b^ See Additional file 2 for complete table showing statistics for all health units

*OR* Odds ratio, *CI* Confidence interval, *r* Rural hospital, *u* Urban hospital, *p* Private hospital, *g* Government hospital

## Discussion

This study showed that the overall prevalence of first trimester orthodox medication consumption among pregnant women in SW Cameroon was 73.2%. When narrowed down to non-pregnancy related orthodox medications, the rate of consumption stood at 68.3%. It is apparent that these prevalences are high compared to those reported in Africa and other developing countries. Out of 1,268 women interviewed during ANC visits in 8 hospitals in Ethiopia, only 29.9% reported at least one drug exposure during the first trimester [12]. This low prevalence of drug exposure compared to the current study could be as a result of recall bias in the Ethiopian study as most women were in their third trimester. Two other studies in Brazil and Pakistan, based on sample sizes of approximately four thousand, reported an overall first trimester exposure prevalence of 22.2% and 11.0% respectively [13, 14]. In the Brazilian study, women were interviewed within 24hrs of delivery. As this period is farther away from the first trimester, coupled with the stress of delivery, it may have been difficult for these women to recall first trimester exposures. The study in Pakistan reviewed only prescriptions given to women during ANC visits - hence over-the-counter medications and medications prescribed elsewhere (e.g. by GP) would have been missed. One study in China [15], based on prospectively collected data reported a similar prevalence (75%) to that seen in our study.

Unlike non-pregnancy related orthodox medications with a high prevalence, first trimester use of pregnancy related orthodox medications in this study was low (42.3%), suggesting underuse of these medications. This observation appears to be true also in other African countries. Based on the entire pregnancy, prevalencesof 42% and 34% have been reported in Nigeria [16] and Ethiopia [12] respectively. Two studies in Nigeria [17] and Tanzania [18] reported higher prevalences (76%, and 94% respectively) of folic acid intake. However, these results were based on exposures across the entire pregnancy and are likely to decrease when restricted to the first trimester as studies in Africa have always reported a far greater proportion (about 40% greater) of medication exposure in the second and third trimesters compared to the first trimester [12, 19]. In our study, 75% of the women registered for ANC after the first trimester. Almost half (46%) of the women booked for ANC late (after 18 weeks), despite WHO recommendations to book by 17 weeks [20], and the women were dependent almost solely on medication given at the hospital. This could help explain the low first trimester intake of pregnancy-related medications which are mainly given during ANC visits.

The most commonly reported group of orthodox medications were analgesics. This seems to corroborate a similar study in Nigeria [16]. As observed in this study and as is the case in sub-Saharan Africa, the intake of analgesics was very high. This could be explained by the high prevalence of infectious diseases in sub- Saharan Africa often associated with fever for which analgesics are indicated. In fact, the intake of analgesics such as paracetamol when one suspects malaria is so common that when the women in our study were asked what paracetamol was indicated for, a few said “it is an accompaniment of antimalarial.”

Anti-anaemias were the most common pregnancy related medications and second most consumed group of medications. Achidi and colleagues (2005) [21] reported that 70% of women registering at their first ANC clinic in SW Cameroon suffer from anaemia. Blood loss is one of the main causes of maternal mortality in sub-Saharan Africa. This is mainly due to infections (mainly malaria) that reduce blood haemoglobin levels and blood loss during delivery (post-partum haemorrhage (PPH)) [22]. Anti-anaemias are, therefore, more highly consumed in sub-Saharan African countries than elsewhere in the world. The observed prevalence (38.6%) of anti-anaemias in this study was, therefore, very low compared to the expected70% prevalence of anaemia. Again, the low intake of anti-anaemias by women in our study could be related to late ANC registration as these drugs are usually prescribed during the ANC visit as prophylaxis.

Sub-Saharan Africa is known to have the largest burden of infectious diseases in the world. It was not surprising, therefore, that anti-infectives were widely used in this study population and that anti-malarials and anti-bacterials were the 3^rd^ and 4^th^ most commonly used orthodox medications by 33.6% and 20.8% of women respectively. However, when compared to the 11-17% prevalence of anti-malarialsinother studies [15, 16, 18] in Nigeria and Tanzania with a similar context to Cameroon, the rates reported here appear to be very high. These studies in Nigeria and Tanzania also reported a 5-15% prevalence of anti-bacterials use. While these studies had a limitation of focusing only in urban areas with possibly lower rates of disease compared to rural areas (although no urban-rural differences in anti-infective use were observed in the current study), the values reported seem surprisingly low compared to the current study.

Sulfadoxine/pyrimethamine (FDA category C), which is contraindicated during the first trimester and which is uniquely indicated as malaria prophylaxis from the second trimester [23], was the most commonly used anti-malarial (13% of women) during the first trimester. Analyses of the data from this study suggest that these drugs were actually used to treat malaria and not taken as preventive. This is in contrast to the observation in Tanzania where, based on the entire pregnancy period, artemether/lumefantrine was the most common antimalarial (8.9% of women) used to treat malaria [18]. However, the Tanzanian study also found that 1.5% of women took Sulfadoxine/pyrimethamine for the treatment of malaria. When used to treat malarial infection, this anti-malarial has a huge potential to develop resistance – a major problem WHO has been struggling to deal with since the drug was first recommended as malaria prophylaxis in pregnancy in 1998 [24]. Given that most of these medications were obtained from the hospital, it is likely that the health personnel are the main contributors to this questionable decision with regard to risk/benefit analysis. There are, of course, many other reasons for this error to occur such as not knowing whether the woman was pregnant, or lack of well trained nurses on the use of these medications. However, one has to be careful about the accuracy of reporting by the women. It is possible that some women reported later use as first trimester; although this problem would not have been unique to Sulfadoxine/pyrimethamine. Important also to highlight is the fact that almost one-third (30%) of women who did not report any malaria illness took a non-prophylactic antimalarial. Indeed, in countries like Cameroon where health care is paid from private pockets, it is common knowledge that patients could be loaded with unnecessary medications in a bid to fill the coffers of the hospital. Nevertheless, it remains a priority to ensure access to antimalarials for all pregnant women as this is essential for their health and survival, and that of their babies.

Most of the women got their orthodox medications mainly from the hospital, as also found in Nigeria [6]. This is an important finding which suggests that safety measures can be particularly targeted at hospitals rather than pharmacies or other sources. However, as indicated in this study, there was a missed opportunity during general consultation when healthcare providers insufficiently prescribed pregnancy related medications and seemingly failed to get the women to register for ANC.

More than two-thirds of women were of the opinion that orthodox medicines are always safe, indicating that women assume safety of medication and professionals need to introduce the element of balancing benefits and risks.

Based on FDA risk classification, it was reassuring to know that women in this study were not exposed to any known teratogenic medications (FDA category X). However, it was noted that up to 17% (excluding women exposed to antimalarials) of women were exposed to at least one FDA category C or D medication (potentially teratogenic).

Our study is the first in Africa to have critically examined a variety of sociodemographic determinants for medication use, and to have divided pregnancy-related and non-pregnancy related medications which were found to have different determinants. Only two studies in Ethiopia have attempted to examine predictors of medication use in pregnancy using a multivariate approach but have major limitations, and it is difficult to compare the results with our own. We found, perhaps not surprisingly, that medication intake is greater where there is illness, but we found that there were determinants of medication intake over and above illness, which suggest differences in behaviour, knowledge or attitudes. This was true of both pregnancy and non-pregnancy related medication. How early the woman booked for ANC was also a strong determinant, particularly of pregnancy related medications. Young maternal age was associated with much lower use of non-pregnancy related medications, but this did not apply specifically to anti-infectives. Women with one or two previous pregnancies were more likely to take non-pregnancy related medications than women in their first pregnancy, or those with three or more previous children, but again this did not apply specifically to anti-infectives.

Health unit was a constant determinant for intake of any of the three categories of orthodox medication suggesting that health units differ in their prescribing behaviour, irrespective of illness status and sociodemographic characteristics of individual women. In Cameroon, private and government hospitals differ in many aspects, including the cost of services (private hospitals being more expensive) and the quality of services (private hospitals giving more attention to patients). Nurses are the highest staff level in health centres, compared to tertiary hospitals which havedoctors and specialists).

Those with a primary education were less likely to take pregnancy related medications; It is possible that the more educated a woman is, the more likely she is to understand why she has to take a medication when she is not sick. However, ability to pay may also play a part. Those who had received safety advice were also more likely to take pregnancy related medications. Perhaps as pregnancy related medications are to be taken for a longer period during pregnancy than most orthodox medications, women need safety advice to reassure them. Women in rural areas were more likely to take pregnancy related medications, perhaps because these medications are highly subsidised in government health centres (primary health units) which are the only hospitals in most rural areas, whereas tertiary health units with less subsidised drugs are in urban areas.

### Strengths and limitations

This study had many strengths as well as limitations. The study is based on interviews with women and, therefore, all medications reported were consumed rather than only prescribed. The sample was designed to be representative of pregnant women in SW Cameroon, with respect both to urban/rural residence, and different types of health facility attended, while other studies in Africa have used less representative samples [12, 14, 15].

Recall bias was limited as the period of interview was mainly within the second trimester. A few women interviewed at the 7^th^ month of gestation reported lower intake of medication, however, this was not significant in multivariate analysis. Additionally, participants’ hospital books were used to complement data reported during interviews and medication picture guides were used to enhance recall. In a validation analysis, it was verified that the hospital books did provide some data which would not have been available based on interviews alone (about 10% of reported medications). Variable availability of hospital books across different health units may have exacerbated differences in medication rates. Although no formal validation was conducted for the use of medication picture guides, the data collectors reported that the picture guides did help the women to recall the medications they took. Even with these measures in place, limitations ofmemory, particularly of short term drug use could not be ruled out. Some women faced the problem of recalling precisely when the first trimester was. And it is also possible that some women just said what they might have thought the interviewer wanted to hear. The use of many data collectors could have introduced interviewer bias. However, the interviewers all went through a standardized training which included pilot testing and validation of the interview process.

Difficulties in dealing with sensitive subjects such as HIV limited the amount of exposure information provided. According to the Cameroon 2011 Demographic Health Survey [25], 5.3% of women are infected with HIV. The rate of 0.2% observed in the current study shows a severe under reporting. Unfortunately, as anti-retrovirals are given as routine medications mainly in HIV treatment centres, information on anti-retroviral exposures are not found in the women’s hospital records. In the Cameroon Prevention of Mother to Child Transmission programme, all HIV positive pregnant women attending ANC are placed on anti-retroviral therapy, but it is difficult to tell if there is any linkage between exposure data at the HIV treatment centres and ANC care units. Therefore, it may have been possible to obtain some data from the ANC files, but there were challenges in accessing these files such as hospital restriction. HIV infection in Cameroon, as is the case with many countries, still carries a stigma. Anti-retrovirals during pregnancy constitute an important topic in pharmacovigilance as the medications have several safety concerns, but are vital during pregnancy to prevent maternal to fetal transmission [26]. Future studies would have to devise means of overcoming the barrier of fear of stigmatisation, such as recruiting the pregnant women during their visit to HIV treatment centres or obtaining permission to access ANC notes.

## Conclusion

Our study confirms the observations of studies across Africa indicating the increasing use of medications during pregnancy, including those that are potentially teratogenic. On the other hand, we also found evidence of under-prescription of medications beneficial for pregnancy. There is an urgent need for public health authorities in Cameroon to put in place medication safety systems targeting pregnant women, especially during the first trimester when the risk of teratogenicity is highest, avoiding unnecessary or contraindicated medication, ensuring that appropriate treatments needed for the health of the pregnant woman and her baby are received, and weighing risks and benefits carefully. Further research is needed about the knowledge and attitudes of health professionals, and pharmacovigilance should track pregnancy outcomes in relation to medication exposures in pregnancy.

## Additional files

Additional file 1: Interview-based Questionnaire. This is the questionnaire developed during and study and used to obtain data from the pregnant women. (DOCX 46 kb)

Additional file 2: These are complete versions of Tables 3, 4 and 5 presented in the text with data for the different health units now included. (DOCX 48 kb)

## Abbreviations

ANC: Antenatal Care
FDA: Food and Drug Administration
GP: General Practitioner
HIV: Human Immunodeficiency Virus
OTC: Over the Counter
PPH: Post Partum Haemorrhage
SW: South West
UK: United Kingdom
WHO: World Health Organisation
