# Supplementary material for: First trimester medication use in pregnancy in Cameroon: a multi-hospital survey
Source: BMC Pregnancy Childbirth. 2018 Nov 20;18:450. doi: 10.1186/s12884-018-2081-x (PMC6245902; doi:10.1186/s12884-018-2081-x)
Supplement: Supplementary file 1 — Interview-based Questionnaire. This is the questionnaire developed during and study and used to obtain data from the pregnant women. (DOCX 46 kb) [file 12884_2018_2081_MOESM1_ESM.docx]

**Project Title:** Maternal medication use during the first trimester in SW Cameroon”

**Project Team:***AminkengLeke; Prof Helen Dolk;Dr.MariaLoane; Dr. Karen Casson,Dr Maboh, MRS. Susan Maeya; Miss Lerry Dibo; Miss Bessem Nyenti;Mr. Armstrong Obale; Mr. Derick Etiendem*

**QUESTIONNAIRE -BASED INTERVIEW**

Hospital Name: ______________________________________________

Date: _______________________

Questionnaire Number________________

PART A: SOCIODEMOGRAPHIC INFORMATION

**Please respond to the following set of questions about your social and demographic information**

**A1**.Age________________

**A2.**Town of permanent residence _________________­­­­­­­­­­­­­­­­­­­­­­­­­­­­­­­_____________________________

**A3.**Marital status

1🞏 Married

2🞏 Divorced

3🞏 Engaged

4🞏 Cohabitating (no formal engagement)

5🞏 Single

**A4.**Highest level of education

1🞏 Never went to school

2🞏 Primary

3🞏 Secondary

4🞏 High school

5🞏 University /Professional

**A5.**Living condition

1🞏 House with pit toilet/external toilet

2🞏 Renting self-contain studio

3🞏 Renting or own a self-contain house

**A6.**Level of alcohol (beer/wine) consumption.

1🞏 Do not drink alcohol

2🞏 Drink occasionally

3🞏 1-2 bottles of beer/ 1glass of wine a week

4🞏 1-2 bottles/1glass of wine a day

5🞏 > 2 bottles/1glass of wine a day

PART B: GYNAECOLOGIC INFORMATION

**Please provide information about your past and present pregnancy**

**B1.**Gravidity (number of pregnancies) ________________

**B2.**Parity________________ NB (consider any live or stillbirth after 24 weeks)

**B3.**Most recent menstruation (LMP)_____________ NB*(woman could verify from ANC record*)

**B4.**Current gestational age___________________

**B5.**Gestationalage at first booking________________NB*(woman could verify from ANC record*)

B6. Was this pregnancy planned?

1🞏 Yes

2🞏 No

B7. Gestational age at which participant became aware of pregnancy status?­­­­­­­­­­­­­­­­­­­­­­­­­­­­­­­­­­­­­­­­­­­­­­­­­­__________________

­­­­­­­­____________________________________

***1^st^ Trimester months:*** 1­­­­­­­­­­­­­­­­­­­­______________________

2______________________

3 ______________________

PART C: INFORMATION ONMEDICATION CONSUMPTION

Medications taken in the first trimester, including those taken intermittently.

**C1.** Orthodox (western, hospital) medications

| S/N | Name of medication | Purchase point (e.g hospital pharmacy (HP), recognised pharmacy (RP), medicine store (MS), retail store (RS)) | Prescriber (e.g doctor, nurse, friend, relative, self) | Indication (Please write **“C”** if woman is certain, **“NC”** if not certain and **“DN”** if she does not know) | Month of pregnancy when treatment was taken (ie 1^st^ 2^nd^ or 3^rd^ ) | Duration (days) of treatment (Please write “**C** “ if subject is still on medication) |
| --- | --- | --- | --- | --- | --- | --- |
| 1 |  |  |  |  |  |  |
| 2 |  |  |  |  |  |  |
| 3 |  |  |  |  |  |  |
| 4 |  |  |  |  |  |  |
| 5 |  |  |  |  |  |  |
| 6 |  |  |  |  |  |  |
| 7 |  |  |  |  |  |  |

*Antibiotics (dosage, date bought and date taken)*

**C2.** Traditional herbs (THM)/ packaged herbal medicine (PHM)***N/B NB: - differentiate between traditional herbs (THM) and packaged herbal medicine (PHM), - please in cases of combinations, list,in brackets,names of all specific herbs within combination.***

| S/N | Name of medication (specific names within combination) | Purchase point (e.g hospital pharmacy (HP), recognised pharmacy (RP), medicine store (MS), retail store (RS)) | Prescriber (e.g doctor, nurse, friend, relative, self) | Indication (Please in write **“C”** if woman is certain, **“NC”** if not certain and **“DN”** if she does not know) | Month of pregnancy when treatment was taken (ie 1^st^ 2^nd^ or 3^rd^ ) | Duration (days) of treatment (Please write “**C** “ if subject is still on medication) |
| --- | --- | --- | --- | --- | --- | --- |
| 1 |  |  |  |  |  |  |
| 2 |  |  |  |  |  |  |
| 3 |  |  |  |  |  |  |

**C3**. Did you discontinue any medications when you realised you were pregnant?

1🞏 Yes, Please list__________________________________________________________________________________________

2🞏 No

PART D: Medical/surgical history

PART D: Medical/surgical history

**The following questions concern illnesses you have had during the first trimester.**

**D1.**Please indicate if you have suffered from any of the following conditions during first three months of pregnancy.

***(NB: Compare information provided with drug information given above and ask follow-up questions accordingly)***

1🞏 Malaria

2🞏 Typhoid

3🞏 Diarrhoea

4🞏 Fever

5🞏 Nausea and vomiting

6🞏 Generalised body pains

7🞏 Cough

8🞏 Catarrh

9🞏 Head ache

10🞏 Others, please list____________________________________________________________

**D2.**Please indicate if you have ever been diagnosed with any of the following chronic conditions.

***(N/B: Compare information provided with drug information given above and ask follow-up questions accordingly)***

1🞏 Diabetes

2🞏 Gestational diabetes

3🞏 Hypertension

4🞏 Arthritis

5🞏 Gastritis

6🞏 Asthma

7🞏 Cancer, please state type__________________________________________

8🞏 Heart disease, please state type__________________________________________

9🞏 Liver disease, please state type__________________________________________

10🞏 Kidney problems, please state type_______________________________________

11🞏 Mental health problem, please state type___________________________________

12🞏 Epilepsy

13🞏 Surgical operation, please state type and reason for operation__________________

14🞏 Others, please list_____________________________________________________

PART E: Other Information on medication utilisation

The following set of questions asks you to give your opinion about taking medications during pregnancy. ***Please remind subject there is no right or wrong answer.***

**E1**.Why did you choose to take herbal medicine instead of /as well as orthodox (western, hospital) medicine? *(****Only for those who took herbal medicine****)*____________________________________________________________

___________________________________________________________________________________________

***I am going to now ask you two questions; after each question I will give you a series of possible answers to choose from. Please choose the answer that best represents your opinion.***

**E2.**Do you think it is safe for a woman to take orthodox (western, hospital) medications during pregnancy?

1🞏 Yes, it is always safe

2🞏 Yes, but it depends…. _____________________________________________

3🞏 No, it is never safe

4🞏 I don't know

**E3.**Do you think it is safe for a woman to take traditional/herbalmedications during pregnancy?

1🞏 Yes, it is always safe

2🞏 Yes, but it depends…._________________________________________

3🞏 No, it is never safe

4🞏 I don't know

**E4.**During this pregnancy has anybody advised/warned you on medication safety during pregnancy?

***(Please list all persons who have given you advice including midwifes, doctors, nurses, family members or friends. NB No Mention of Names!)***

1🞏 Yes, by whom_________________________________________________________________

2🞏 No

3🞏 Can’t remember

**Thank you for your participation in this study.**
