# Supplementary material for: First trimester medication use in pregnancy in Cameroon: a multi-hospital survey
Source: BMC Pregnancy Childbirth. 2018 Nov 20;18:450. doi: 10.1186/s12884-018-2081-x (PMC6245902; doi:10.1186/s12884-018-2081-x)
Supplement: Supplementary file 2 — These are complete versions of Tables 3, 4 and 5 presented in the text with data for the different health units now included. (DOCX 48 kb) [file 12884_2018_2081_MOESM2_ESM.docx]

**APPENDIX 2**

**Complete Table 3,4 and 5**

**Table 3:** Logistic regression model for predicting consumption of non-pregnancy related orthodox medication (N=795)

|  | **Crude model** | | | | | | | | **Adjusted model** | | | | | |
| --- | --- | --- | --- | --- | --- | --- | --- | --- | --- | --- | --- | --- | --- | --- |
| **Variables** | **Exposed**  **(%)** | **OR** | | **95% C.I** | | | | **P-value** | **OR** | **95% C.I** | | | | **P-value** |
|  |  |  |  | Lower | | Upper | |  |  | Lower | | Upper | |  |
| **Health unit** | | | | | | | | 0.000^¥^ |  | | | | | 0.000^¥^ |
| A^r,p^ | 44.2 | 1 | | - | | - | | - | 1 | - | | - | | ref |
| B^r,g^ | 81.1 | 5.4 | | 2.0 | | 15.0 | |  | 10.9 | 2.1 | | 57.5 | |  |
| C^r,g^ | 65.5 | 2.4 | | 0.9 | | 6.4 | |  | 4.5 | 0.8 | | 25.7 | |  |
| D^r,g^ | 86.4 | 8.0 | | 2.1 | | 31.1 | |  | 3.8 | 0.6 | | 24.3 | |  |
| E^u,p^ | 93.1 | 17.1 | | 3.6 | | 80.9 | |  | 21.6 | 1.6 | | 285.4 | |  |
| F^u,g^ | 59.0 | 1.8 | | 0.8 | | 4.4 | |  | 1.3 | 0.3 | | 5.0 | |  |
| G^r,g^ | 47.2 | 1.1 | | 0.5 | | 2.8 | |  | 3.7 | 0.9 | | 15.5 | |  |
| H^r,g^ | 71.4 | 3.2 | | 1.2 | | 8.2 | |  | 3.3 | 0.8 | | 13.4 | |  |
| I^r,g^ | 73.1 | 3.4 | | 1.2 | | 9.8 | |  | 0.9 | 0.2 | | 3.6 | |  |
| J^u,g^ | 70.5 | 3.0 | | 1.2 | | 7.3 | |  | 4.3 | 1.2 | | 15.8 | |  |
| K^u,p^ | 88.0 | 9.3 | | 3.3 | | 26.3 | |  | 5.4 | 1.3 | | 22.5 | |  |
| L^u,g^ | 80.0 | 5.1 | | 1.2 | | 20.5 | |  | 6.3 | 0.9 | | 44.5 | |  |
| M^u,g^ | 70.1 | 3.0 | | 1.3 | | 6.6 | |  | 8.4 | 2.3 | | 30.9 | |  |
| N^u,p^ | 94.4 | 21.5 | | 2.6 | | 176.2 | |  | 11.1 | 1.0 | | 126.4 | |  |
| O^r,g^ | 39.8 | 0.8 | | 0.4 | | 1.7 | |  | 1.0 | 0.3 | | 3.1 | |  |
| P^r,g^ | 85.7 | 7.6 | | 1.9 | | 29.6 | |  | 14.9 | 2.7 | | 82.3 | |  |
| Q^r,g^ | 88.9 | 10.1 | | 3.3 | | 30.6 | |  | 11.2 | 2.3 | | 55.3 | |  |
| R^r,g^ | 64.3 | 2.3 | | 1.0 | | 5.1 | |  | 3.6 | 1.0 | | 13.7 | |  |
| S^r,g^ | 69.4 | 2.9 | | 1.2 | | 6.7 | |  | 2.2 | 0.6 | | 8.5 | |  |
| T^u,g^ | 88.5 | 9.7 | | 2.5 | | 37.2 | |  | 44.2 | 4.0 | | 483.6 | |  |
| **Maternal age (years)** | | | | | | | | 0.095^¥^ |  | | | | | 0.049^¥^ |
| 18-25 | 71.3 | | 1 | | - | | - | - | 1 | | - | | - | ref |
| 13-17 | 53.7 | | 0.5 | | 0.2 | | 0.9 |  | 0.1 | | 0.0 | | 0.8 |  |
| 26-35 | 66.3 | | 0.8 | | 0.6 | | 1.1 |  | 0.6 | | 0.4 | | 1.1 |  |
| 36-45 | 71.8 | | 1.0 | | 0.5 | | 2.1 |  | 1.3 | | 0.5 | | 3.8 |  |
| **Parity** | | | | | | | | 0.107^¥^ |  | | | | | 0.006^¥^ |
| 0 | 68.7 | | 1 | | - | | - | - | 1 | | - | | - | ref |
| 1 | 70.8 | | 1.1 | | 0.8 | | 1.6 |  | 2.9 | | 1.0 | | 8.6 |  |
| 2 | 71.7 | | 1.2 | | 0.8 | | 1.8 |  | 4.0 | | 1.3 | | 12.7 |  |
| ≥3 | 59.2 | | 0.7 | | 0.4 | | 1.0 |  | 1.5 | | 0.5 | | 4.9 |  |
| **Gestational age at ANC booking** | | | | | | | | 0.000^¥^ |  | | | | | 0.001^¥^ |
| 13 - 24 weeks | 65.8 | | 1 | | - | | - | - | 1 | | - | | - | ref |
| < 13 weeks | 83.9 | | 2.7 | | 1.8 | | 4.1 |  | 2.6 | | 1.3 | | 5.2 |  |
| 25 - 28 weeks | 52.2 | | 0.6 | | 0.2 | | 1.3 |  | 1.6 | | 0.4 | | 6.4 |  |
| Not registered for ANC | 36.4 | | 0.3 | | 0.2 | | 0.6 |  | 0.3 | | 0.1 | | 0.8 |  |
| **Illness during first trimester** | | | | | | | | 0.000^¥^ |  | | | | | 0.000^¥^ |
| No | 32.8 | | 1 | | - | | - | - | 1 | | - | | - | ref |
| Yes | 79.4 | | 7.9 | | 5.5 | | 11.3 |  | 11.1 | | 6.0 | | 20.4 |  |
| **Opinion on safety of orthodox medication** | | | | | | | | 0.180^¥^ |  | | | | | 0.078^¥^ |
| Yes, it is always safe | 67.2 | | 1 | | - | | - | - | 1 | | - | | - | ref |
| Yes, safe but depends | 75.2 | | 1.5 | | 1.0 | | 2.2 |  | 0.7 | | 0.4 | | 1.3 |  |
| No, never safe | 66.7 | | 1.0 | | 0.4 | | 2.5 |  | 0.1 | | 0.0 | | 0.8 |  |
| I don't know | 62.1 | | 0.8 | | 0.5 | | 1.4 |  | 0.5 | | 0.2 | | 1.3 |  |

*¥ Overall statistical significance of variable within model*

*OR, Odds ratio; CI, Confidence interval*

*r: Rural hospital; u: Urban hospital; p:Private hospital; g: Government hopsital*

**Table 4:** Logistic regression model for predicting consumption of pregnancy related orthodox medication (N=795)

|  | **Crude model** | | | | | | | | | | | | | | **Adjusted model** | | | | | | | | | |
| --- | --- | --- | --- | --- | --- | --- | --- | --- | --- | --- | --- | --- | --- | --- | --- | --- | --- | --- | --- | --- | --- | --- | --- | --- |
| **Variables** | **Exposed**  **(%)** | **OR** | | | | **95% C.I** | | | | | | | | **P-value** | **OR** | **95% C.I** | | | | | | | | **P-value** |
|  |  |  |  |  |  | Lower | | | | Upper | | | |  |  | Lower | | | | Upper | | | |  |
| **Health unit** | | | | | | | | | | | | | | 0.000^¥^ |  | | | | | | | | | 0.000^¥^ |
| A^r,p^ | 32.6 | 1 | | | | - | | | | - | | | | - | 1 | - | | | | - | | | | ref |
| B^r,g^ | 35.1 | 1.1 | | | | 0.4 | | | | 2.8 | | | |  | 1.1 | 0.3 | | | | 3.9 | | | |  |
| C^r,g^ | 58.6 | 2.9 | | | | 1.1 | | | | 7.8 | | | |  | 3.2 | 0.8 | | | | 13.1 | | | |  |
| D^r,g^ | 81.8 | 9.3 | | | | 2.7 | | | | 32.8 | | | |  | 7.1 | 1.5 | | | | 34.4 | | | |  |
| E^u,p^ | 55.2 | 2.5 | | | | 1.0 | | | | 6.7 | | | |  | 4.5 | 0.9 | | | | 22.6 | | | |  |
| F^u,g^ | 25.6 | 0.7 | | | | 0.3 | | | | 1.9 | | | |  | 1.3 | 0.2 | | | | 6.5 | | | |  |
| G^r,g^ | 27.8 | 0.8 | | | | 0.3 | | | | 2.1 | | | |  | 1.0 | 0.3 | | | | 3.8 | | | |  |
| H^r,g^ | 48.6 | 2.0 | | | | 0.8 | | | | 4.9 | | | |  | 2.9 | 0.9 | | | | 9.4 | | | |  |
| I^r,g^ | 38.5 | 1.3 | | | | 0.5 | | | | 3.6 | | | |  | 1.7 | 0.5 | | | | 6.3 | | | |  |
| J^u,g^ | 40.9 | 1.4 | | | | 0.6 | | | | 3.4 | | | |  | 5.8 | 1.3 | | | | 26.2 | | | |  |
| K^u,p^ | 76.0 | 6.6 | | | | 2.6 | | | | 16.3 | | | |  | 13.5 | 2.7 | | | | 68.1 | | | |  |
| L^u,g^ | 53.3 | 2.4 | | | | 0.7 | | | | 7.8 | | | |  | 5.4 | 0.9 | | | | 30.7 | | | |  |
| M^u,g^ | 44.8 | 1.7 | | | | 0.8 | | | | 3.7 | | | |  | 5.7 | 1.3 | | | | 25.2 | | | |  |
| N^u,p^ | 72.2 | 5.4 | | | | 1.6 | | | | 18.1 | | | |  | 5.9 | 0.7 | | | | 47.3 | | | |  |
| O^r,g^ | 13.9 | 0.3 | | | | 0.1 | | | | 0.8 | | | |  | 1.2 | 0.2 | | | | 5.8 | | | |  |
| P^r,g^ | 47.6 | 1.9 | | | | 0.6 | | | | 5.5 | | | |  | 4.3 | 0.8 | | | | 22.8 | | | |  |
| Q^r,g^ | 57.8 | 2.8 | | | | 1.2 | | | | 6.8 | | | |  | 5.5 | 1.8 | | | | 17.0 | | | |  |
| R^r,g^ | 21.4 | 0.6 | | | | 0.2 | | | | 1.4 | | | |  | 0.9 | 0.3 | | | | 2.7 | | | |  |
| S^r,g^ | 57.1 | 2.8 | | | | 1.2 | | | | 6.5 | | | |  | 8.0 | 1.6 | | | | 38.8 | | | |  |
| T^u,g^ | 50.0 | 2.1 | | | | 0.8 | | | | 5.6 | | | |  | 4.7 | 0.9 | | | | 25.0 | | | |  |
| **Highest level of Education** | | | | | | | | | | | | | | 0.000^¥^ |  | | | | | | | | | 0.072^¥^ |
| Secondary | 37.4 | | 1 | | | | - | | | | - | | | - | 1 | | - | | | | - | | | ref |
| Never went to school | 38.0 | | 1.0 | | | | 0.7 | | | | 1.5 | | |  | 0.9 | | 0.5 | | | | 1.4 | | |  |
| Primary | 26.3 | | 0.6 | | | | 0.2 | | | | 1.7 | | |  | 0.2 | | 0.0 | | | | 0.6 | | |  |
| High School | 45.0 | | 1.4 | | | | 0.9 | | | | 2.1 | | |  | 1.0 | | 0.5 | | | | 1.7 | | |  |
| University/Professional | 63.7 | | 2.9 | | | | 1.9 | | | | 4.6 | | |  | 1.4 | | 0.7 | | | | 3.0 | | |  |
| **Gestational age at ANC booking** | | | | | | | | | | | | | | **-** |  | | | | | | | | | 0.004^¥^ |
| 13 - 24 weeks | 39.5 | | | 1 | | | | - | | | | - | | 0.000^¥^ | 1 | | | - | | | | - | | ref |
| < 13 weeks | 59.8 | | | 2.3 | | | | 1.6 | | | | 3.2 | |  | 1.4 | | | 0.9 | | | | 2.4 | |  |
| 25 - 28 weeks | 8.7 | | | 0.1 | | | | 0.0 | | | | 0.6 | |  | 0.4 | | | 0.1 | | | | 1.9 | |  |
| Not registered for ANC | 13.6 | | | 0.2 | | | | 0.1 | | | | 0.6 | |  | 0.2 | | | 0.1 | | | | 0.6 | |  |
| **Participant received safety advice** | | | | | | | | | | | | | | 0.001^¥^ |  | | | | | | | | | 0.057^¥^ |
| Yes | 47.6 | | | 1 | | | | - | | | | - | | - | - | | | - | | | | - | | ref |
| No | 34.5 | | | 0.6 | | | | 0.4 | | | | 0.8 | |  | 0.6 | | | 0.4 | | | | 0.9 | |  |
| Can't remember | 35.0 | | | 0.6 | | | | 0.3 | | | | 1.2 | |  | 0.7 | | | 0.2 | | | | 2.1 | |  |
| **Setting type** | | | | | | | | | | | | | | 0.609^¥^ |  | | | | | | | | | 0.042^¥^ |
| Urban | 41.5 | | | | 1 | | | | - | | | | - | - | 1 | | | | - | | | | - | ref |
| Rural | 43,3 | | | | 1.1 | | | | 0.8 | | | | 1.4 |  | 3.3 | | | | 1.0 | | | | 10.2 |  |

*¥ Overall statistical significance of variable within model*

*OR, Odds ratio; CI, Confidence interval*

*r: Rural hospital; u: Urban hospital; p:Private hospital; g: Government hopsital*

**Table 5:** Logistic regression model for predicting consumption of anti-infectives (N=795)

|  | **Crude model** | | | | | | | | | | | | | | | | | **Adjusted model** | | | | | | | | | |
| --- | --- | --- | --- | --- | --- | --- | --- | --- | --- | --- | --- | --- | --- | --- | --- | --- | --- | --- | --- | --- | --- | --- | --- | --- | --- | --- | --- |
| **Variables** | **Exposed**  **(%)** | | | | **OR** | | | | **95% C.I** | | | | | | | | **P-value** | **OR** | **95% C.I** | | | | | | | | **P-value** |
|  |  |  |  |  |  |  |  |  | Lower | | | | Upper | | | |  |  | Lower | | | | Upper | | | |  |
| **Health unit** | | | | | | | | | | | | | | | | | 0.000^¥^ |  | | | | | | | | | 0.001^¥^ |
| A^r,p^ | 32.6 | | | | 1 | | | | - | | | | - | | | | - | 1 | - | | | | - | | | | ref |
| B^r,g^ | 45.9 | | | | 1.8 | | | | 0.7 | | | | 4.4 | | | |  | 5.1 | 1.3 | | | | 19.9 | | | |  |
| C^r,g^ | 20.7 | | | | 0.5 | | | | 0.2 | | | | 1.6 | | | |  | 0.6 | 0.1 | | | | 3.0 | | | |  |
| D^r,g^ | 59.1 | | | | 3.0 | | | | 1.0 | | | | 8.7 | | | |  | 2.7 | 0.6 | | | | 11.8 | | | |  |
| E^u,p^ | 69.0 | | | | 4.6 | | | | 1.7 | | | | 12.7 | | | |  | 4.9 | 1.0 | | | | 24.5 | | | |  |
| F^u,g^ | 51.3 | | | | 2.2 | | | | 0.9 | | | | 5.3 | | | |  | 2.7 | 0.8 | | | | 9.7 | | | |  |
| G^r,g^ | 25.0 | | | | 0.7 | | | | 0.3 | | | | 1.9 | | | |  | 1.1 | 0.2 | | | | 5.4 | | | |  |
| H^r,g^ | 34.3 | | | | 1.1 | | | | 0.4 | | | | 2.8 | | | |  | 2.2 | 0.6 | | | | 7.6 | | | |  |
| I^r,g^ | 50.0 | | | | 2.1 | | | | 0.8 | | | | 5.6 | | | |  | 1.5 | 0.4 | | | | 5.9 | | | |  |
| J^u,g^ | 45.5 | | | | 1.7 | | | | 0.7 | | | | 4.1 | | | |  | 4.1 | 1.4 | | | | 12.6 | | | |  |
| K^u,p^ | 80.0 | | | | 8.3 | | | | 3.2 | | | | 21.3 | | | |  | 8.5 | 2.4 | | | | 30.2 | | | |  |
| L^u,g^ | 66.7 | | | | 4.1 | | | | 1.2 | | | | 14.4 | | | |  | 5.9 | 1.3 | | | | 27.0 | | | |  |
| M^u,g^ | 46.3 | | | | 1.8 | | | | 0.8 | | | | 4.0 | | | |  | 4.3 | 1.4 | | | | 12.7 | | | |  |
| N^u,p^ | 77.8 | | | | 7.3 | | | | 2.0 | | | | 26.1 | | | |  | 9.1 | 1.3 | | | | 63.7 | | | |  |
| O^r,g^ | 25.0 | | | | 0.7 | | | | 0.3 | | | | 1.5 | | | |  | 1.5 | 0.5 | | | | 4.1 | | | |  |
| P^r,g^ | 33.3 | | | | 1.0 | | | | 0.3 | | | | 3.1 | | | |  | 1.2 | 0.3 | | | | 4.9 | | | |  |
| Q^r,g^ | 62.2 | | | | 3.4 | | | | 1.4 | | | | 8.2 | | | |  | 5.3 | 1.7 | | | | 16.7 | | | |  |
| R^r,g^ | 37.5 | | | | 1.2 | | | | 0.5 | | | | 2.9 | | | |  | 2.9 | 0.9 | | | | 9.3 | | | |  |
| S^r,g^ | 32.7 | | | | 1.0 | | | | 0.4 | | | | 2.4 | | | |  | 1.4 | 0.4 | | | | 4.6 | | | |  |
| T^u,g^ | 73.1 | | | | 5.6 | | | | 1.9 | | | | 16.5 | | | |  | 9.4 | 2.3 | | | | 38.6 | | | |  |
| **Gestational age at ANC booking** | | | | | | | | | | | | | | | | | 0.00^¥^ |  | | | | | | | | | 0.000^¥^ |
| 13 - 24 weeks | | 42.2 | | | | 1 | | | | - | | | | - | | | - | 1 | | - | | | | - | | | ref |
| < 13 weeks | | 62.8 | | | | 2.3 | | | | 1.7 | | | |  | | |  | 2.7 | | 1.6 | | | | 4.5 | | |  |
| 25 - 28 weeks | | 17.4 | | | | 0.3 | | | | 0.1 | | | |  | | |  | 0.6 | | 0.1 | | | | 2.4 | | |  |
| Not registered for ANC | | 11.4 | | | | 0.2 | | | | 0.1 | | | |  | | |  | 0.3 | | 0.1 | | | | 0.9 | | |  |
| **Illness during first trimester** | | | | | | | | | | | | | | | | | | | | | | | | | | | |
| No | | | 20.6 | | | | - | | | | - | | | | - | | - | - | | | - | | | | - | | ref |
| Yes | | | 52.5 | | | | 4.2 | | | | 2.9 | | | | 6.3 | | 0.000^¥^ | 3.7 | | | 2.1 | | | | 6.4 | | 0.000^¥^ |
| **Opinion on safety of orthodox medication** | | | | | | | | | | | | | | | | | 0.098^¥^ |  | | | | | | | | | 0.056^¥^ |
| Yes, it is always safe | | | | 46.6 | | | | 1 | | | | - | | | | - | - | 1 | | | | - | | | | - | ref |
| Yes, safe but depends | | | | 45.9 | | | | 1.0 | | | | 0.7 | | | | 1.4 |  | 0.6 | | | | 0.4 | | | | 1.1 |  |
| No, never safe | | | | 33.3 | | | | 0.6 | | | | 0.2 | | | | 1.4 |  | 0.2 | | | | 0.0 | | | | 1.2 |  |
| I don't know | | | | 31.8 | | | | 0.5 | | | | 0.3 | | | | 0.9 |  | 0.4 | | | | 0.2 | | | | 1.0 |  |

*¥ Overall statistical significance of variable within model*

*OR, Odds ratio; CI, Confidence interval*

*r: Rural hospital; u: Urban hospital; p:Private hospital; g: Government hospital*
